# Clinical performance of medical students in Korea in a whole-task emergency station in the objective structured clinical examination with a standardized patient complaining of palpitations

**DOI:** 10.3352/jeehp.2020.17.42

**Published:** 2020-12-16

**Authors:** Song Yi Park, Hyun-Hee Kong, Min-Jeong Kim, Yoo Sang Yoon, Sang-Hwa Lee, Sunju Im, Ji-Hyun Seo

**Affiliations:** 1 Department of Emergency Medicine, Dong-A University College of Medicine, Busan, Korea; 2 Department of Medical Education, Dong-A University College of Medicine, Busan, Korea; 3 Department of Parasitology, Dong-A University College of Medicine, Busan, Korea; 4 Department of Medical Education and Neurology, Kosin University College of Medicine, Busan, Korea; 5 Department of Emergency Medicine, Inje University College of Medicine, Busan, Korea; 6 Department of Medical Education, Pusan National University School of Medicine, Busan, Korea; 7 Department of Pediatrics, Gyeongsang National University School of Medicine, Jinju, Korea; 8 Department of Medical Education, Gyeongsang National University School of Medicine, Jinju, Korea; 9 Gyeongsang National Institute of Health Sciences, Jinju, Korea; Hallym University, Korea

**Keywords:** Medical student, Electrocardiography, Clinical competence, Emergency medicine, Republic of Korea

## Abstract

This study assessed the clinical performance of 150 third-year medicalstudents in Busan, Korea in a whole-task emergency objective structured clinical examination station that simulated a patient with palpitations visiting the emergency department. The examination was conducted from November 25 to 27, 2019. Clinical performance was assessed as the number and percentage of students who performed history-taking (HT), a physical examination (PE), an electrocardiography (ECG) study, patient education (Ed), and clinical reasoning (CR), which were items on the checklist. It was found that 18.0% of students checked the patient’s pulse, 51.3% completed an ECG study, and 57.9% explained the results to the patient. A sizable proportion (38.0%) of students did not even attempt an ECG study. In a whole-task emergency station, students showed good performance on HT and CR, but unsatisfactory results for PE, ECG study, and Ed. Clinical skills educational programs for subjected student should focus more on PE, timely diagnostic tests, and sufficient Ed.

## Background/rationale

Palpitations are a common clinical presentation in patients who visit the emergency department (ED). The etiology of palpitations ranges from benign factors to life-threatening cardiac problems. Electrocardiography (ECG) is a non-invasive and widely applicable diagnostic tool that can be used at the patient’s bedside [1]. Students’ competency in performing structured and fast patient assessments and performing ECG studies should be acquired and assessed during undergraduate medical education.

In the clinical skills portion of the Korean Medical License Examination (KMLE), palpitations are considered to be 1 of the 54 clinical presentations of the standardized patient (SP) encountered during the objective structured clinical examination (OSCE). Furthermore, ECG is 1 of the 32 procedural skills that are tested (Supplement 1). In general, SPs can simulate symptoms of palpitations, but cannot reproduce abnormal physical examination or ECG results. Thus, the skills required during encounters with an SP (history-taking, physical examination, diagnostic workup planning) and performing ECG studies were previously evaluated as separate tasks in isolation. However, in real clinical contexts in the ED, the physician takes the patient’s history and performs a physical examination and ECG study simultaneously to differentiate the cause of palpations as an integrated process. Some studies have described the advantages of whole-task OSCEs, which simulate integrated processes in real-world clinical practice [2,3]. However, no studies have reported medical students’ clinical performance on a whole-task emergency case OSCE station.

## Objectives

Our research question was, “How will medical students perform in a whole-task emergency case station as part of an OSCE with an SP who complains of palpitations?” The purpose of this study was to examine the clinical performance of third-year medical students in a whole-task emergency case OSCE station that simulated a patient visiting the ED due to sustained palpitations. The results will be able to provide medical teachers the information on how to help students in preparation for the clinical skills test of the KMLE

## Ethics statement

This study was approved by the institutional review board of Dong-A University (IRB approval no., 2-1040709-AB-N-01-202002-HR-003-02). The requirement for informed consent was waived because subjects participated in the examination as a scheduled part of the educational curriculum.

## Study design

This was a cross-sectional observational study.

## Participants

A total of 150 third-year medical students who attended the first day of the Busan-Gyeongnam Clinical Skill Examination (BGCSE), which was summative and mandatory, were included in this study. Students were from 5 universities in Busan: Dong-A University, Gyeongsang National University, Kosin University, Inje University, and Pusan National University.

## Setting

The BGCSE Consortium is an association of 5 medical schools in the Busan-Gyeongnam region in South Korea that have conducted joint clinical skill examinations for the OSCE for 3rd-year medical students annually since 2014. In 2019, a whole-task emergency case OSCE station was developed by faculty members from 5 medical schools, including 2 emergency physicians, 1 pediatrician, 1 neurologist, and 2 medical education experts who attended 5 half-day workshops. The examination included 11 other traditional OSCE stations (6 SP encounters and 5 technical skills) at the skills centers of 4 medical schools in Busan (Dong-A University, Kosin University, Inje University, and Pusan National University) from November 25 to 27, 2019. The students were allowed 10 minutes at each station, and a 5-minute interval was allowed between stations.

The scenario involved a 28-year-old man with palpitations. He reported having occasional palpitations, which usually lasted for approximately 10 minutes, and they suddenly started and abruptly ended. He visited the ED after the palpitations lasted for an hour, which was considered unusual, earlier that day.

The SPs were trained by an SP trainer about the scenarios for 2 hours and rehearsed for standardization for 2 more hours. All the SPs had more than 5 years of SP experience for the BGCSE. Four experienced faculty members from 4 medical schools in Busan volunteered to be examiners. Briefings about the scoring rubrics, how to mark the scores using the computer program, and the importance of confidentiality were conducted at the orientation held for the examiners.

**Fig. 1.**
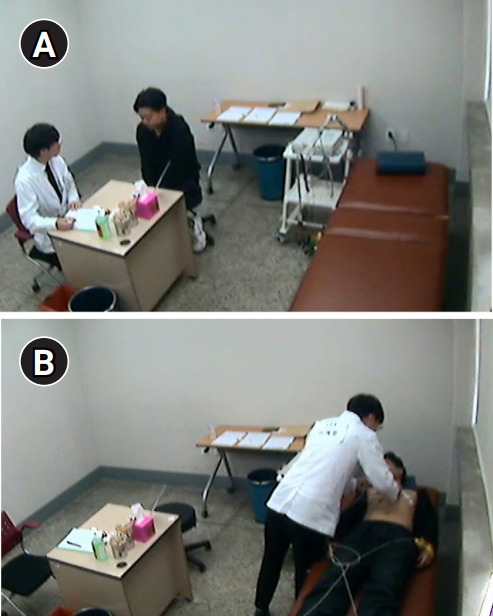
(A) A student taking the history of an standardized patient (SP). (B) A student performing an electrocardiography study with the SP on the bed. Informed consent was obtained from the student and SP.

The setting of the station is shown in Fig. 1. The SP was first interviewed by the student and then laid on the bed for a physical examination and ECG study. ECG electrodes were placed on the SP by a student, and an assistant provided the prepared normal ECG results. The students were informed about the whole-task station at their BGCSE orientation.

## Measurement

The instructions provided to the students (outside the station) were as follows: “You are the primary physician in the ED; you are expected to take a history from this patient; perform a focused physical examination; select an appropriate diagnostic tool and perform the evaluation; develop a treatment plan with the patient and educate the patient accordingly; and write down the most probable diagnosis after the station is completed.”

The checklist consisted of 5 categories: history-taking (13 binary items), physical examination (5 items scored on a 3-point scale), ECG study (6 items including both binary and 3-point scale items), patient education (3 binary items), and clinical reasoning (1 item with an open-ended written question). The global rating scale included 2 items (scored on a 5-point scale): the identification of the SP before the ECG study and the student’s proficiency. There were no standards regarding passing/failing scores for this station because there were no data that could be used for reference. The clinical performance was presented as the number and percentage of students who successfully performed history-taking, a physical examination, an ECG study, patient education, and clinical reasoning according to the items on the checklist.

## Statistical methods

Descriptive statistics including the number and percentage of students who performed the clinical skills corresponding to the checklist items were used to determine the clinical performance. All variables were analyzed using an Excel spreadsheet (Microsoft Corp., Redmond, WA, USA).

## Clinical performance for history-taking and the physical examination

For history-taking, the performance rate was the highest for checklist item 3 (98.7%; checking whether the patient had a previous history of palpitations) and the lowest for checklist item 9 (23.3%; checking whether the patient’s palpitations had ever been accompanied by a loss of consciousness) (Table 1). In the physical examination, 18.0% of students checked the patient’s pulse (Table 2). Raw data are available from Dataset 1.

## Clinical performance for the ECG study

Ninety-three (62.0%) students tried an ECG study, while 57 (38.0%) students did not even attempt an ECG study (Table 3).

## Clinical performance for patient education and clinical reasoning

Forty-two (28.0%) students explained the ECG results to the patient (Table 4). Of the 69 students who completed the ECG study, 40 (57.9%) explained the ECG results to the SP. Almost all (98%) of the students reported paroxysmal supraventricular tachycardia as the most likely diagnosis.

## Key results

This study examined the clinical performance of 3rd-year medical students in a whole-task emergency case OSCE station where an ECG study was performed for an SP complaining of palpitations in a simulated ED. The main finding is that a third of students did not attempt an ECG study and focused on taking a detailed patient history and performing a non-emergent physical examination to rule out non-cardiac causes of palpitations, even though the ECG equipment was prepared next to the patient’s bed (Fig. 1).

## Interpretation

The key goal of this whole-task case was to check whether the patient had palpitations at the time of presentation and to differentiate potentially fatal arrhythmia first. Students listened to the patient's detailed and non-urgent medical history, as was the case with the existing SP encounter partial task. This is mostly likely because we previously provided separate education and evaluation of the SP encounter skill (palpitations) and procedural skill (ECG study). Students likely also had the basic assumption that the SP would not be able to simulate palpitations at the OSCE station. The fact that students can do partial tasks does not seem to guarantee that they can do the whole task. Furthermore, it seems that clinical skills need to be acquired with abundant clinical context. While this study did not compare whether the OSCE design (whole versus partial task) affected students’ learning strategies, the whole-task OSCE appears to be advantageous for structuring pre-assessment learning.

## Comparison with previous studies

To the best of our knowledge, we could not find any previous studies on the ECG performance rate of medical students in whole-task emergency case OSCEs. Few studies about emergency case OSCEs have been reported [4], and in a previous report, the ECG study was designed as a partial-task station in an emergency case OSCE composed of 6 whole-task stations and 4 traditional technical-skill stations [5]. In a hybrid station that tried a whole-task OSCE combining an SP encounter and a simulated Papanicolaou test for a clinical skills examination, the station was found to be highly authentic, acceptable, and feasible [6]. However, that study compared a new station with a traditional one, and did not report the students’ clinical performance. According to studies on whole-task OSCEs, whole-task stations increase students’ use of diagnostic reasoning skills during the study period [2]. Whole-task OSCEs might make students use different learning strategies from those used to approach partial- and fixed-task OSCE stations, which lead to the compartmentalization of certain skills for the examination [3,7]. Cilliers et al. [8] proposed these tendencies and educational effects in a model of the pre-assessment learning effect.

## Limitations

First, only 1 case may not be enough to assess students’ clinical performance. The medical students’ clinical performance in this study cannot be inferred as reflecting their future competence in a workplace [9]. Second, the responses of the students and examiners in this study were not investigated in depth. Third, 10 minutes may not have been long enough for students to examine the SP. Fourth, some students may have performed an ECG study only because they noticed that the ECG equipment was ready at the station.We did not assess how many students would have planned an ECG study if the equipment had not been available at the test station.

## Conclusion

Few studies have assessed whole-task emergency-case OSCE stations performed by medical students. The clinical performance of 3rd-year medical students at a whole-task emergency station was fair regarding history-taking and clinical reasoning, but unsatisfactory regarding the physical examination, ECG study, and patient education. It is necessary to strengthen physical examination, procedural skills, and patient education competencies in preparation for the clinical skills test of the KMLE.

## Figures and Tables

**Table 1.** Clinical performance of the 3rd-year medical students regarding taking the history of the standardized patient with palpitations

No.	Checklist	Clinical performance of 150 students	
		Yes (%)	No (%)
1	I found that there were no palpitations now.	88 (58.9)	62 (41.1)
2	I found that the palpitations began about an hour ago.	145 (96.7)	5 (3.3)
3	I found that the patient had experienced a few short palpitations in the past.	148 (98.7)	2 (1.3)
4	I found that the patient had palpitations once or twice a year.	70 (46.7)	80 (53.3)
5	I found that the palpitations lasted about an hour.	93 (62.0)	57 (38.0)
6	I found that palpitations occurred suddenly while resting after lunch.	103 (68.7)	47 (31.3)
7	I found that the palpitations began suddenly.	108 (72.0)	42 (28.0)
8	I found that the palpitations ended suddenly.	77 (51.3)	73 (48.7)
9	I found that the patient never lost consciousness during palpitations.	35 (23.3)	115 (76.7)
10	I found that the patient had no history of heart disease or heart surgery.	63 (42.0)	87 (58.0)
11	I found that no sudden weight loss or heat intolerance had occurred recently.	74 (49.3)	76 (50.7)
12	I found that the patient took no medications.	134 (89.3)	16 (10.7)
13	I found that no extreme anxiety or fear accompanied the palpitations.	42 (28.0)	108 (72.0)

**Table 2.** Clinical performance of the 3rd-year medical students regarding performing a physical examination for the standardized patient with palpitations

No.	Checklist	Clinical performance of 150 students		
		Performed (%)	Partially performed (%)	Not performed (%)
1	Performed a conjunctival examination.	97 (64.7)	3 (2.0)	50 (33.3)
2	Checked the pulse.	27 (18.0)	6 (4.0)	117 (78.0)
3	Performed heartbeat auscultation.	71 (47.3)	42 (28.0)	37 (24.7)
4	Performed lung sound auscultation.	19 (12.7)	36 (24.0)	95 (63.3)
5	Palpated the thyroid gland.	48 (32.0)	23 (15.3)	79 (52.7)

**Table 3.** Clinical performance of the 3rd-year medical students for the ECG study of the standardized patient with palpitations

No.	Checklist	Clinical performance of 150 students		
		Performed (%)	Partially performed (%)	Not performed (%)
1	Explained to patient the need for an ECG test.	73 (48.7)	-	77 (51.3)
2	Wiped the patient’s skin with an alcohol swab for the ECG test.	75 (50.0)	-	75 (50.0)
3	Attached standard limb electrodes to the patients at the correct locations.	90 (60.0)	-	60 (40.0)
4	Identified at least 1 intercostal space when attaching the chest electrode.	50 (33.3)	-	100 (66.7)
5	Attached standard chest electrodes to the patients at the correct locations.	77 (51.3)	16 (10.7)	57 (38.0)
6	Asked the patient not to move before conducting the ECG test.	47 (31.3)	-	103 (68.7)
7	Overall ECG study	77 (51.3)	16 (10.7)	57 (38.0)

ECG, electrocardiography.

**Table 4.** Clinical performance of the 3rd-year medical students regarding patient education

No.	Checklist	Clinical performance of 150 students	
		Performed (%)	Not performed (%)
1	Explained the electrocardiography results to the patient.	42 (28.0)	108 (72.0)
2	Educated the standardized patient to revisit the emergency department if the palpitations recur.	36 (24.0)	114 (76.0)
3	Explained to the patient that coffee or alcohol can cause palpitations.	30 (20.0)	120 (80.0)

## Footnotes

**Data availability** Data files are available from Harvard Dataverse: https://doi.org/10.7910/DVN/FHWPHK Dataset 1. Raw data of the students’ responses and the result data.

## Supplementary materials

Data files are available from Harvard Dataverse: https://doi.org/10.7910/DVN/FHWPHK

Supplement 1. Korean Medical License Examination clinical skill test topics.

Supplement 2. Audio recording of the abstract.
