# Supplementary material for: Clinical performance of medical students in Korea in a whole-task emergency station in the objective structured clinical examination with a standardized patient complaining of palpitations
Source: J Educ Eval Health Prof. 2020 Dec 16;17:42. doi: 10.3352/jeehp.2020.17.42 (PMC7856094; doi:10.3352/jeehp.2020.17.42)
Supplement: Supplementary file 2 — Supplement 1. Korean Medical License Examination clinical skill test topics. [file jeehp-17-42-suppl1.pdf]

Supplement 1. Korean Medical License Examination clinical skill test topics

	Topics	
**Standardized patient encounter**	1. Easy bruise	2. Sleep disturbance
	3. Anxiety	4. Alcohol abuse and dependence
	5. Mood change	6. Grave news consultation
	7. Domestic violence	8. Drug abuse
	9. Chest pain	10. Syncope
	11. Palpitation	12. High blood pressure
	13. Cough	14. Dyspnea
	15. Hemoptysis	16. Rhinorrhea/nose stuffy
	17. Abdominal pain	18. Dyspepsia
	19. Melena	20. Constipation
	21. Diarrhea	22. Hematemesis
	23. Jaundice	24. Vomiting
	25. Breast mass and pain	26. Dyslipidemia
	27. Polyuria	28. Oliguria
	29. Weight gain	30. Joint pain
	31. Back pain	32. Neck pain
	33. Abnormal urination	34. Red urine
	35. Urinary frequency	36. Vaccination
	37. Fatigue	38. Fever
	39. Weigh loss	40. Smoking consultation
	41. Antenatal care	42. Vaginal discharge
	43. Abnormal menstruation	44. Growth/development delay
	45. Seizure	46. Headache
	47. Altered mentality	48. Extremity weakness and sensory change
	49. Tremor	50. Decline in cognition
	51. Skin rash	52. Dizziness
	53. Suicide attempt	54. Sexual violence

**Procedure skills**	1. Chest X-ray presentation	2. Blood sampling for culture
	3. Venous blood sampling	4. Arterial blood sampling
	5. Cerebrospinal fluid sampling	6. Papanicolaou test
	7. Wet smear test	8. Electrocardiography study
	9. Otoscopic examination	10. Fundus examination
	11. Blood pressure examination	12. Digital rectal examination
	13. Splinting of musculoskeletal injuries	14. Basic life support
	15. Primary closure	16. Peripheral venous access
	17. Transfusion	18. Injection (intradermal, subcutaneous, intramuscular)
	19. Wound dressing	20. Burn dressing
	21. Local anesthesia	22. Incision and drainage abscess
	23. Management of normal labor	24. Cardiac electric shock therapy
	25. Urinary catheterization	26. Tracheal intubation
	27. Foreign body airway obstruction	28. Informed consent
	29. Communication of medical information	30. Visual acuity examination
	31. Surgical gowning and gloving	32. Endotracheal suction
